# Nur77 Mediates Anaphylaxis by Regulating miR-21a

**DOI:** 10.3390/cimb46040199

**Published:** 2024-04-06

**Authors:** Hyein Jo, Jaewhoon Jeoung, Kyeonghee Shim, Dooil Jeoung

**Affiliations:** Department of Biochemistry, Kangwon National University, Chuncheon 24341, Republic of Korea; qnfdudn1212@gmail.com (H.J.); heyjhw@kangwon.ac.kr (J.J.); sim991127@kangwon.ac.kr (K.S.)

**Keywords:** allergic inflammation, C-JUN, miR-21a, Nur77

## Abstract

Nur77 belongs to the NR4A subfamily of orphan nuclear hormone receptors. It has been shown to play important roles in metabolism, cancer progression, cellular differentiation, and the regulation of immune process. However, there has yet to be research reporting on the role of Nur77 in allergic inflammations such as anaphylaxis. This study aimed to identify molecules that could mediate allergic inflammations. To this end, we performed RNA sequencing analysis employing bone marrow-derived mast cells (BMMCs). Antigen (DNP-HSA) stimulation increased the expression levels of transcription factors such as Nr4a3 (NOR1), Nr4a1 (Nur77), and Nr4a2 (Nurr1). We focused our study on Nur77. Antigen stimulation increased the expression of Nur77 in a time- and dose-dependent manner in rat basophilic leukemia cells (RBL2H3). The downregulation of Nur77 prevented both antigen-induced increase in β-hexosaminidase activity as well as hallmarks of allergic reactions such as HDAC3, COX2, and MCP1 in RBL2H3 cells. Nur77 was necessary for both passive cutaneous anaphylaxis (PCA) and passive systemic anaphylaxis (PSA). TargetScan analysis predicted that miR-21a would be a negative regulator of Nur77. miR-21a mimic negatively regulated PCA and PSA by inhibiting the hallmarks of allergic reactions. ChIP assays showed that c-JUN could bind to the promoter sequences of Nur77. Antigen stimulation increased the expression of c-JUN in RBL2H3 cells. Altogether, our findings demonstrate the regulatory role played by Nur77-miR-21a loop in allergic inflammations such as anaphylaxis, making this the first report to present the role played by Nur77 in an allergic inflammation. Our results suggest that Nur77 and miR-21 might serve as targets for developing anti-allergy drugs.

## 1. Introduction

Transcription factor Nur77 belongs to the nuclear receptor subfamily 4 group A (NR4A) [1]. Nur77 transcription factor is expressed as an early response gene [2]. Nur77 can bind to CXC motif chemokine receptor 4 (CXCR4) to promote glioma invasion [1]. CXCR4-CXC motif chemokine ligand 12 (CXCL12) signaling can promote allergic airway remodeling [3]. The increased expression of Nur77 has been shown to be necessary for cigarette smoke-induced lung inflammation [4]. Nur77 has also been shown to mediate lipopolysaccharide-induced inflammation by activating nuclear factor-kB (NF-kB) in a mouse model of acute lung injury [5].

Allergic inflammation involves cellular interactions [6,7]. The polarization of M2 macrophages occurs during the progression of allergic inflammations [6,7,8]. Nur77 can promote M2 polarization by increasing the number of interleukin-10 (IL-10) producing CD206^+^ macrophages [9]. 

Anaphylaxis is known to be closely associated with enhanced angiogenic potential [10,11]. Nur77 can mediate angiogenesis by increasing the expression of integrin β, which in turn activates Phosphoinositide 3-kinase (PI3K)/Akt/Focal adhesion kinase (FAK) signaling [12]. Nur77 can also attenuate endothelial dysfunction by increasing nitric oxide production [13].

The acetylation of Nur77 has been shown to enhance the stability of Nur77 [14]. Histone deacetylase 1 (HDAC1) decreases the expression and transcriptional activity of Nur77 [14]. In previous work, we reported that HDAC3 [6] and HDAC6 [7] mediate allergic inflammations. 

Nur77 can mediate allergic airway remodeling by promoting autophagy [15]. Autophagy promotes allergic inflammations by mediating cellular interactions [8]. IL-25, which is increased by allergic airway inflammation, can promote the polarization of M2 macrophages by stimulating autophagy [16]. 

Taken together, these reports suggest that Nur77 plays an essential role in allergic inflammations such as anaphylaxis. In the present study, we found that Nur77 was highly upregulated by antigen stimulation in bone marrow-derived mast cells (BMMCs). Nur77, which is a member of the NR4A subfamily, was found to be necessary for both in vitro allergic reactions and anaphylaxis. Micro RNA-21a (miR-21a) was found to function as a negative regulator of Nur77. Moreover, we showed that miR-21a plays an inhibitory role in anaphylaxis. Altogether, the findings of our study suggest that the Nur77-miR-21a loop can function as a target for developing anti-allergy drugs. 

## 2. Materials and Methods

### 2.1. Materials

We purchased chemicals from Sigma-Aldrich (St. Louis, MO, USA). Horseradish peroxidase (HRP)-conjugated goat anti-rabbit IgG was purchased from ENZO Life science (# ADI-SAB-300-J, New York, NY, USA). HRP-conjugated goat anti-mouse IgG was purchased from Cell signaling (# 7076, Danvers, MA, USA). We purchased primers from Bioneer Company (Daejeon, Republic of Korea). The sequences of mimics are listed in Supplementary Table S1.

### 2.2. Animals

Female BALB/C mice (~20 g, 8 weeks) were purchased from Nara Biotech (Seoul, Republic of Korea). Animals were kept under standard housing conditions (20~26 °C, 150~300 lux, 40~60% humidity) with 14-10 hour light–dark period. Animals were allowed free access to food and water. All procedures were conducted in accordance with the ARRIVE guidelines. All animal experiments were conducted according to the guidelines of the Korean Council for the Care and Use of Animals in Research and approved by the Institutional Animal Care and Use Committee (IACUC) of the Kangwon National University (KW-190425-2). Animal euthanasia was performed using CO_2_ gas at 30–70% displacement rate of the cage volume/min using a flow meter according to the American Veterinary Medical Association (AVMA) euthanasia guideline 43.

### 2.3. Cell Culture

RBL2H3 cells were purchased from the ATCC (Manassas, VA, USA). Cells were grown in Dulbecco’s modified Eagle’s medium containing heat-inactivated fetal bovine serum, 2 mM L-glutamine, 100 unit/mL penicillin, and 100 μg/mL streptomycin (Invitrogen, Waltham, MA, USA). Cultures were maintained in 5% CO_2_ at 37 °C. Cells were tested using a e-Myco^TM^ plus Mycoplasma PCR Detection Kit (iNtRON, Seongnam, Republic of Korea, cat # 25237) to ensure that they were mycoplasma free. Bone marrow-derived mast cells (BMMCs) were isolated according to the standard procedures with slight modifications [8]. BMMCs were isolated from the femoral and tibia bone marrow cells of BALB/C mice. BMMCs were grown in Dulbecco’s modified Eagle’s medium (DMEM) containing heat-inactivated fetal bovine serum (10%) and IL-3 (30 ng/mL). Cell number and viability were determined using trypan blue staining. The β-hexosaminidase activity was determined to check the functionality of BMMCs. We routinely check the expression of FcεRI. 

### 2.4. RNA Sequencing and Analysis

TRIzol^®^ RNA Isolation Reagents (FAVORGEN BIOTECH, Chung-Chem 1st Rd. Kaohsiung, Taiwan) were used for the extraction of total RNA. Messenger RNA sequencing library was constructed by using the Illumina TruSeq Stranded mRNA Sample Preparation kit (Illumina). All libraries were quantified by qPCR (CFX96, Bio-Rad, Hercules, CA, USA) and sequenced on the NextSeq500 sequencers (Illumina) with a paired-end 75 bp plus single 8 bp index read run. To quantify the mapped reads on the reference genome into the gene expression values, Cuffquant in Cufflinks with the strand-specific library option and other default options was used. The differentially expressed genes were analyzed by Cuffdiff software (version 0.8.0) with the strand-specific library option [17]. To compare the expression profiles among the samples, the normalized expression values of a few hundred of the differentially expressed genes were clustered by in-house R scripts. The heatmap of the expression values of the selected DEGs in log10 (FPKM) units was compared across genes and samples (fold changes > 2 and *p*-value < 0.05). GO and KEGG enrichment analyses were performed by g:Profiler2 ver. 0.2.

### 2.5. Data Availability

The RNA seq. data sets can be found at the NCBI’s Sequence Read Archive (https://www.ncbi.nlm.nih.gov/bioproject/787734, accessed on 10 December 2021) (PRJNA787734).

### 2.6. Transfections

Small interfering RNAs (SiRNAs) and microRNA (miR) mimics were purchased from Bioneer Company (Daejeon, Republic of Korea). For transfections, JetPEI^®^ (Polyplus, New York, NY, USA, cat.201-10G) was used. Transfections were performed according to the manual provided by the manufacturer (Polyplus, New York, NY, USA). The sequences of siRNAs are shown in Supplementary Table S2. RBL2H3 cells were transfected with siRNA (each at 10 nM) or miR mimic (each at 10 nM) for 24 h. At 24 h after seeding, cells were transfected with JetPEI^®^ (Polyplus, cat.201-10G). All transfections were performed in the presence of serum. 

### 2.7. Quantitative Real Time PCR

Total microRNA (miRNA) was isolated by miRNeasy Mini Kit (QIAGEN, Hilden, Germany). CDNA synthesis from miRNA was carried out according to the manual provided by the manufacturer (Sigma-Aldrich, St. Louis, MO, USA). miR-21a expression level was determined based on the threshold (Ct), and relative expression level was determined as 2-(Ct of miR-21a-5p)-(Ct of U6) after normalization to the expression of U6 small nuclear RNA. For quantitative real-time PCR (QRT-PCR), SYBR PCR Master Mix (Applied Biosystems, Waltham, MA, USA) was used in a StepOne TM Real-Time System (ThermoFisher, Cat# 4376357, Waltham, MA, USA). Supplementary Table S3 shows the primer sequences used in this study.

Total RNA was isolated from tissues or cells by using TRIzol reagent (Thermo Fisher, Waltham, MA, USA). One μg total RNA was converted into cDNA using a pre-mix reverse transcription kit (iNtRon Biotechnology, Kyunggi, Republic of Korea). QRT-PCR was performed using the synthesized cDNA and a SYBR-green mixture containing the Rox dye (Excel Taq™ 2X Fast Q-PCR Master Mix) (SMOBIO, Hsinchu, Taiwan) in a StepOne^TM^ Real-Time PCR System (Thermo Fisher). The PCR conditions were 40 cycles of denaturation for 30 s at 95 °C, annealing for 30 s at 60 °C, and extension for 30 s at 72 °C. Supplementary Table S3 shows the primer sequences. Relative mRNA levels were determined using the ΔΔCt value and normalized to that of mouse or rat actin mRNA.

### 2.8. ELISA

The serum histamine level was determined using an ELISA kit (Abcam, Cambridge, UK). ELISA was performed according to the standard procedure provided by the manufacturer (Abcam, Cat# 213975). In brief, samples were added to wells coated with a goat anti-rabbit IgG antibody. Anti-histamine antibody and biotin-histamine tracer were added to the wells. After incubation and washing, Horseradish Peroxidase-conjugated Streptavidin (SA-HRP) was added to all wells and the plate was incubated. After incubation, TMB (3,3′,5,5′-Tetramethylbenzidine) substrate was added to the wells and the plate was incubated. Stop solution was added. The resulting yellow color was read at 450 nm.

### 2.9. Chromatin Immunoprecipitation Assay

Chromatin immunoprecipitation (ChIP) assays were performed according to the standard procedures [8]. In brief, the RBL2H3 cells were cross-linked in 4% formaldehyde solution for 10 min, and ChIP DNA was isolated. Lysates were extracted and chromatin was sheared by sonication to 400–500 bp, followed by centrifugation to remove cell debris. Primary antibody for actin (2 μg/mL) or c-Jun (2 μg/mL) was incubated at 4 °C for 16 h. Immunoprecipitation was performed with Protein A/G PLUS-Agarose (Santacruz, sc-2003) at 4 °C for 14 h. PCR was performed with specific primers of the Nur77 promoter-1 (5′-CCTGTTTTCTGATGCTCCTAGG-3′ (sense) and 5′-CCCACTGCCAGCAAGAAG-3′(antisense)), Nur77 promoter-2 (5′-TTCATAACACCAAGCTGGGTTG-3′ (sense) and 5′-GACTTGCAGCTCTAACACTCAC-3′ (antisense)), and Nur77 promoter-3 (5′-CCCTCCTCTTGGCCCATATT-3′ (sense) and 5′-AAGGCTCTCTTGGCTAGGA-3′ (antisense)) sequences to determine the binding of protein of interest (c-JUN). PCR was performed in a MiniAmp™ Plus Thermal Cycler (ThermoFisher, Cat#A37835). The PCR conditions were 40 cycles of denaturation for 30 s at 95 °C, annealing for 30 s at 58 °C, and extension for 30 s at 72 °C.

### 2.10. Immunoblot and Immunoprecipitation

Cells were lysed in lysis buffer (50 mM Tris-HCl (pH, 6.8), 150 mM NaCl, 1% NP-40, 50 mM dithiothreitol) supplemented with 1 mM sodium ortho vanadate, 1% protease inhibitor cocktail (Roche, Switzerland). Lysates were cleared by centrifugation (15,000× *g*, 4 °C, 15 min) and protein concentrations were quantified with Bicinchoninic Acid (BCA) Protein Assay Kit (GenDEPOT, Katy, TX, USA). Cell lysates were separated on 10% sodium dodecyl sulfate-polyacrylamide gel electrophoresis and then transferred onto polyvinylidene difluoride (PVDF) membrane. Following blocking in TBS-T with 2% BSA for 1 h, the membranes were then incubated with the indicated primary antibodies overnight at 4 °C. After washing three times in TBS-T, membranes were incubated with the respective secondary antibody for 2 h. After washing with TBS-T, immunodetection was performed using West-Q Pico ECL Solution (GenDEPOT, Katy, TX, USA). Chemiluminescence images were obtained using Amersham™ ImageQuant™ 500 imaging system (Cytiva, Washington, DC, USA). The following primary antibodies were used: Nur77 (Proteintech, 12235-1-AP, Chicago, IL, USA), HDAC3 (Cell Signaling, #3949, Danvers, MA, USA), COX2 (Cell Signaling, #12282), MCP1 (Abcam, ab25124), FcεRIβ (sc-393789, Santa Cruz, CA, USA), Lyn (sc-7274, Santa Cruz, CA, USA), pLyn^Y507^ (Cell Signaling, 2731, Danvers, MA, USA), and Actin (Proteintech, #66009).

To isolate tissue lysates, tissue was frozen in liquid nitrogen and homogenized with RIPA buffer. After vortexing and centrifugation at 10,000× *g* for 15 min at 4 °C, supernatant was used as tissue lysates. 

### 2.11. Passive Cutaneous Anaphylaxis

BALB/C mice were given an intradermal injection with DNP-IgE (0.5 μg/kg) along with intravenous injection with control mimic (3 μg/kg) or miR-21a mimic (3 μg/kg). The next day, BLAB/C mice were intravenously injected with PBS or 2,4-dinitrophenyl-human serum albumin (DNP-HSA) (250 μg/kg) along with 2% (*v*/*v*) Evans blue solution. To determine the effect of Nur77 on the PCA, BALB/C mice were given an intradermal injection with DNP-IgE (0.5 μg/kg) along with an intravenous injection with negative control siRNA (3 μg/kg) or Nur77 siRNA (3 μg/kg).

### 2.12. Passive Systemic Anaphylaxis

BALB/C mice were intravenously injected with DNP-specific IgE (0.5 μg/kg) along with miR-21a mimic or control mimic (3 μg/kg). Twenty-four hours later, BALB/C mice were intravenously injected with PBS or DNP-HSA (250 μg/kg). Rectal temperatures were measured. To determine the effect of Nur77 on the PSA, BALB/C mice were intravenously injected with DNP-specific IgE (0.5 μg/kg) along with control siRNA or Nur 77 siRNA (3 μg/kg). Twenty-four hours later, BALB/C mice were given an intravenous injection with PBS or DNP-HSA (250 μg/kg).

### 2.13. Statistical Analysis

Data were analyzed by using the GraphPad Prism statistics program (GraphPad Prism Ver.7 software). The data are presented as means ± S.E. One-way ANOVA was carried out for comparisons among three or more groups and was followed by Tukey’s range test. *p*-values *<* 0.05 were considered to be statistically significant.

## 3. Results

### 3.1. Nur77 Mediates In Vitro Allergic Reactions

**Figure 1:**
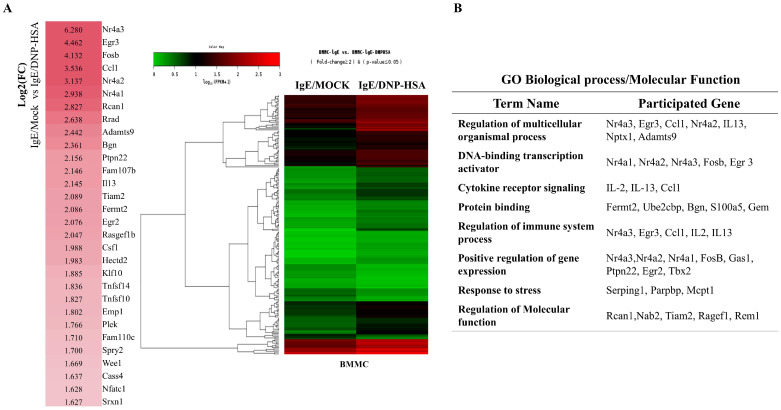
Identification of genes that are regulated by antigen in bone marrow-derived mast cells. (**A**) BMMCs were sensitized with DNP-specific IgE (100 ng/mL) for 24 h. Cells were then stimulated with DNP-HSA (100 ng/mL) for 1 h. RNA sequencing analysis was carried out by employing total RNAs. The heat map of the expression values of the selected DEGs in log10 (FPKM) units was compared across genes and samples (fold changes > 2 and *p*-value < 0.05). (**B**) Gene ontology analysis of highly upregulated genes.

**Figure 2:**
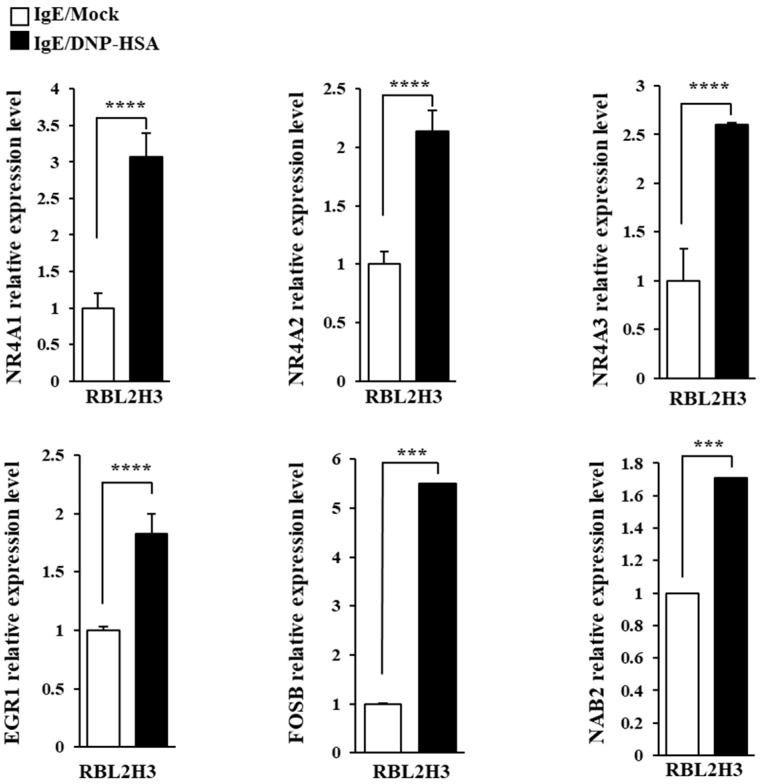
QRT-PCR confirmation of RNA sequencing analysis. The IgE-sensitized RBL2H3 cells were stimulated with DNP-HSA (100 ng/mL) for 1 h. QRT-PCR was performed as described. The means ± S.E. of three independent experiments are shown. One-way ANOVA was carried out. ***, *p* < 0.001; ****, *p* <0.0001.

**Figure 3:**
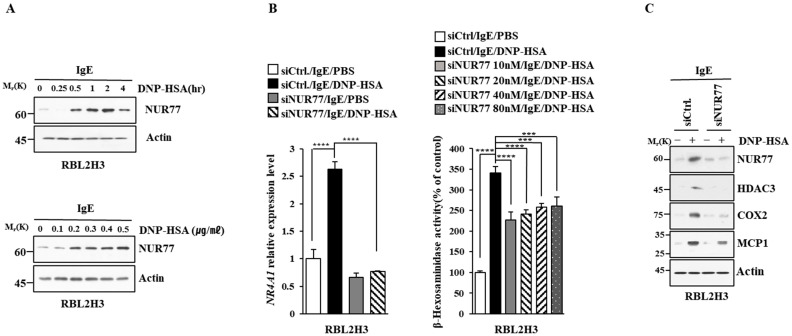
Nur77 mediates in vitro allergic reactions. (**A**) The IgE-sensitized RBL2H3 cells were stimulated with DNP-HSA (100 ng/mL) for various time intervals (upper panel). The IgE-sensitized RBL2H3 cells were stimulated with various concentrations of DNP-HSA for 1 h (lower panel). Representative blots of three independent experiments are shown. The uncropped blots are shown in Supplementary Materials. (**B**) At 24 h after transfection with the indicated siRNA, cells were sensitized with DNP-specific IgE (100 ng/mL) for another 24 h, followed by stimulation with DNP-HSA (100 ng/mL) for 1 h. SiCtrl. (80 nM) denotes the negative control siRNA. QRT-PCR and β-hexosaminidase activity assays were performed. The means ± S.E. of three independent experiments are shown. One-way ANOVA was carried out. ***, *p* < 0.001; ****, *p* < 0.0001. (**C**) At 24 h after transfection with the indicated siRNA (each at 10 nM), cells were then sensitized with DNP-specific IgE (100 ng/mL) for 24 h, followed by stimulation with DNP-HSA (100 ng/mL) for 1 h. Representative blots of three independent experiments are shown. The uncropped blots are shown in Supplementary Materials.

We first identified molecules that are regulated by antigen in RBL2H3 cells, as we believe that these molecules would play essential roles in allergic inflammations. To this end, we conducted RNA sequencing analyses employing bone marrow-derived mast cells (BMMCs) from BALB/C mice (Figure 1A). Nr4a1 (Nur77), Nr4a2 (Nurr1), and Nr4a3 (Nor1) were found to be highly upregulated by antigen stimulation (Figure 1A). Neuron-derived orphan receptor (Nor1) plays an essential role in the proliferation and migration of airway smooth cells [18]. Early growth response 3 (Egr3) and NGF-1-A binding protein 2 (Nab2) were also found to be highly upregulated by antigen stimulation in BMMCs (Figure 1A). Egr2 and Egr3 can induce the expression of Nab2 in melanoma cells [19]. Egr2 plays an important role in asthma by regulating Th17 cell differentiation [20]. TH2 cytokines have been shown to mediate allergic inflammations [21]. Antigen stimulation was shown to increase the expression of IL-13 in BMMCs (Figure 1A). FOSB was shown to induce the expression of tumor necrosis factor (TNF) in antigen-stimulated mast cells [22]. FOSB was found to be upregulated by antigen stimulation in BMMCs (Figure 1A). Prior research has found that patients with asthma show high expression levels of FosB and Egr3 [23]. Genes that are highly upregulated by antigen stimulation play important roles in various life processes, including transcription, cytokine signaling, the regulation of immune processes, the regulation of molecular function, and stress response (Figure 1B). Quantitative real-time PCR (QRT-PCR) showed that the expression levels of Nur77, Nurr1, and Nor1 were indeed increased by antigen simulation in rat basophilic leukemia (RBL2H3) cells (Figure 2). Antigen stimulation was found to increase the expression of NUR77 in a dose- and time-dependent manner in RBL2H3 cells (Figure 3A). Antigen stimulation also increased the expression of Nur77 at the transcriptional level (Figure 3B). The downregulation of Nur77 exerted a negative effect on the increase in β-hexosaminidase activity in response to antigen stimulation in RBL2H3 cells (Figure 3B). The downregulation of Nur77 also prevented antigen from increasing the hallmarks of allergic reactions such as HDAC3, cyclooxygenase 2 (COX2), and monocyte chemoattractant protein 1 (MCP1) (Figure 3C). MCP1 is known to mediate atopic dermatitis [24] and anaphylaxis [7,8]. Nur77 can protect against experimental colitis by decreasing NF-kB activity, which in turn decreases the expressions of both MCP1 and CXCL1 in macrophages [25]. This implies that Nur77 might play a regulatory role in allergic inflammations. Mast cell degranulation is accompanied by the activation of PI3K-AKT-NF-kB signaling [26]. This indicates that Nur77 may activate PI3K-AKT-NF-kB signaling. Altogether, these results suggest that Nur77 plays a role in allergic reactions. 

### 3.2. Nur77 Mediates Anaphylaxis

**Figure 4:**
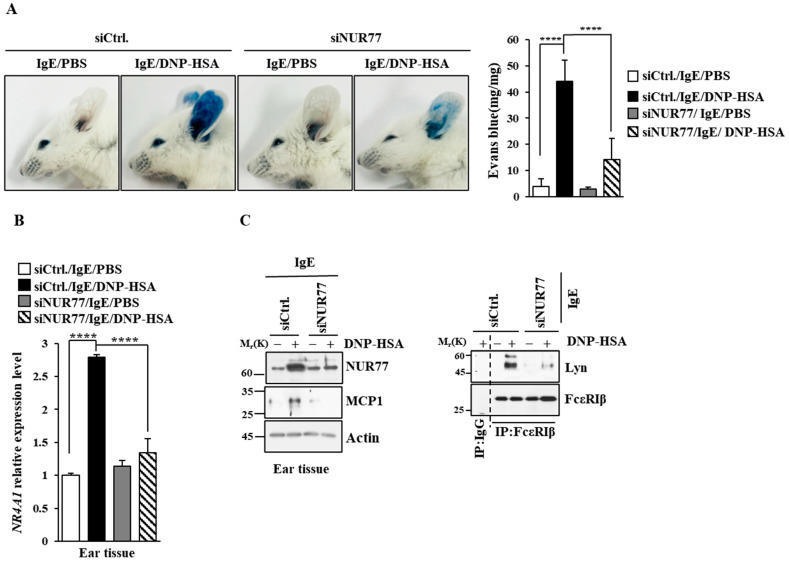
Nur77 mediates PCA. (**A**) BALB/C mice were given an intradermal injection of DNP-specific IgE (0.5 mg/kg) and the indicated siRNA (each at 3 μg/kg) was intravenously injected. The following day, mice were given an intravenous injection of PBS or DNP-HSA (250 μg/kg) with 2% (*v*/*v*) Evans blue solution. Each experimental group comprised four BALB/C mice. The means ± S.E. of three independent experiments are shown. One-way ANOVA was carried out. ****, *p* < 0.0001. (**B**) Ear tissue lysates were subjected to qRT-PCR. The means ± S.E. of three independent experiments are shown. One-way ANOVA was carried out. ****, *p* < 0.0001. (**C**) Immunoblot and immunoprecipitation employing ear tissue lysates were performed. Representative blots of three independent experiments are shown. Immunoprecipitation using isotype-matched IgG is shown. The uncropped blots are shown in Supplementary Materials.

**Figure 5:**
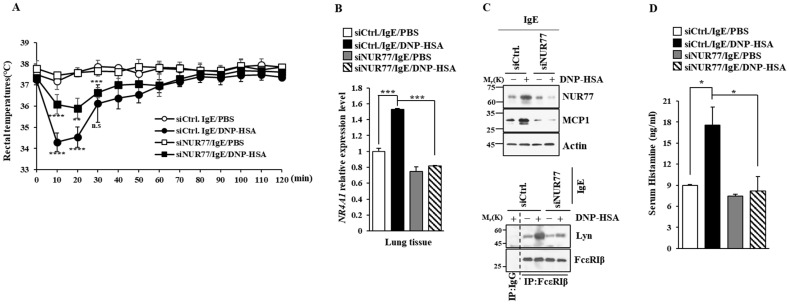
Nur77 mediates PSA. (**A**) BALB/C mice were given an intravenous injection with the indicated siRNA (each at 3 μg/kg). The next day, mice were given an intravenous injection of DNP-specific IgE (0.5 mg/kg). The next day, mice were subjected to an intravenous injection with DNP-HSA (250 μg/kg), and their rectal temperatures were measured. Each experimental group comprised five mice. The means ± S.E. of three independent experiments are shown. One-way ANOVA was carried out. *, *p* < 0.05; **, *p* < 0.01, comparison was made between SiCtrl./IgE/DNP-HSA and SiNUR77/IgE/DNP-HSA. ****, *p* < 0.0001, comparison was made between SiCtrl./IgE/PBS and SiCtrl./IgE/DNP-HSA. n.s. denotes not significant. (**B**) QRT-PCR employing cell lysates was performed. The means ± S.E. of three independent experiments are shown. One-way ANOVA was carried out. ***, *p* < 0.001. (**C**) Immunoblot and immunoprecipitation employing cell lysates were performed. Representative blots of three independent experiments are shown. Immunoprecipitation using isotype-matched IgG is shown. The uncropped blots are shown in Supplementary Materials. (**D**) Serum histamine level was determined as described. The means ± S.E. of three independent experiments are shown. One-way ANOVA was carried out. *, *p* < 0.05.

Next, we investigated the role that Nur77 plays in anaphylaxis. Nur77 was found to be necessary for PCA (Figure 4A). PCA was found to increase vascular permeability in a Nur77-depdent manner in a mouse model of PCA (Figure 4A). PCA also increased Nur77 expression at the transcriptional level (Figure 4B). Immunoblot of ear tissue lysates showed that NUR77 was necessary for the increased expression of MCP1 in a mouse model of PCA (Figure 4C). The binding of FcεRI to LYN occurred in a Nur77-dependent manner in a mouse model of PCA (Figure 4C). Ovalbumin-induced PCA has been shown to be mediated by thymic stromal lymphopoietin (TSLP) [27]. Thus, Nur77 may mediate PCA by increasing the expression of TSLP. PSA decreased rectal temperatures in a Nur77-dependent manner (Figure 5A). Peanut-induced food allergy has been shown to induce changes in body temperature and histamine level [28]. PSA was found to increase Nur77 expression at the transcriptional level (Figure 5B). Nur77 was also necessary for the increased expression of MCP1 and the binding of FcεRI to LYN in a mouse model of PSA (Figure 5C). Nur77 was also necessary for achieving an increased serum histamine level in a mouse model of PSA (Figure 5D). Therefore, Nur77 appears to play an essential role in anaphylaxis. 

### 3.3. miR-21a and miR-124 Negatively Regulates In Vitro Allergic Reactions 

**Figure 6:**
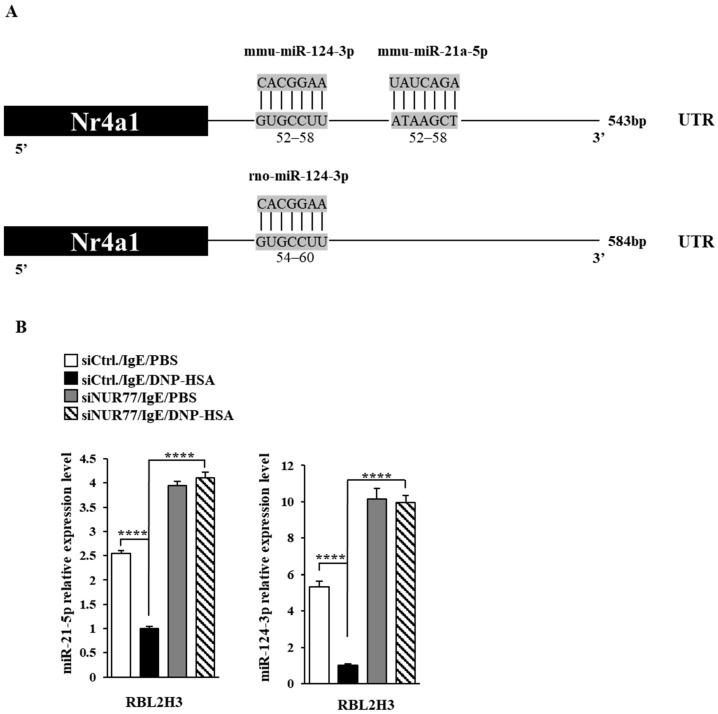
Nur77 negatively regulates the expressions of miR-21a and miR-124. (**A**) miRNAs that can bind to the 3′UTR of Nur77. (**B**) At 24 h after transfection with the indicated siRNA (each at 10 nM), cells were sensitized with DNP-specific IgE (100 ng/mL) for another 24 h, followed by stimulation with DNP-HSA (100 ng/mL) for 1 h. SiCtrl. denotes the negative control siRNA. QRT-PCR was performed. One-way ANOVA was carried out. ****, *p* < 0.0001.

**Figure 7:**
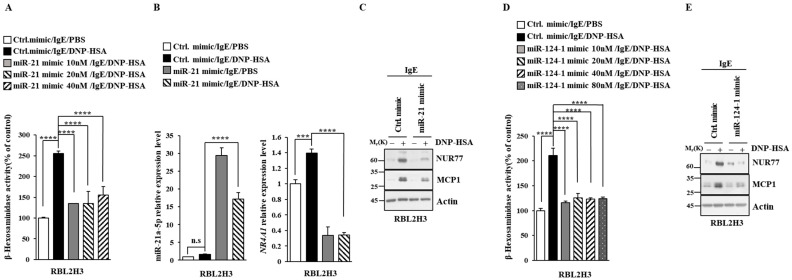
miR-21a suppresses in vitro allergic reactions. (**A**) At 24 h after transfection with the indicated miR-mimic, cells were sensitized with DNP-specific IgE (100 ng/mL) for another 24 h, followed by stimulation with DNP-HSA (100 ng/mL) for 1 h. Ctrl. mimic denotes control mimic. β-hexosaminidase activity assay was performed. One-way ANOVA was carried out. ****, *p* < 0.0001. (**B**) At 24 h after transfection with the indicated mimic (each at 10 nM), cells were sensitized with DNP-specific IgE (100 ng/mL) for another 24 h, followed by stimulation with DNP-HSA (100 ng/mL) for 1 h. Ctrl. mimic denotes control mimic. QRT-PCR was performed. One-way ANOVA was carried out. ***, *p* < 0.001; ****, *p* < 0.0001. n.s. denotes not significant. (**C**) Immunoblot was performed. Representative blots of three independent experiments are shown. The uncropped blots are shown in Supplementary Materials. (**D**) The indicated mimic, at the indicated concentration, was transfected into RBL2H3 cells. At 24 h after transfection, cells were sensitized with DNP-specific IgE (100 ng/mL) for another 24 h, followed by stimulation with DNP-HSA (100 ng/mL) for 1 h. β-hexosaminidase activity assay was performed. The means ± S.E. of three independent experiments are shown. One-way ANOVA was carried out. ****, *p* < 0.0001. (**E**) At 24 h after transfection with the indicated mimic (each at 10 nM), cells were sensitized with DNP-specific IgE (100 ng/mL) for another 24 h, followed by stimulation with DNP-HSA (100 ng/mL) for 1 h. Immunoblot was performed. Representative blots of three independent experiments are shown. The uncropped blots are shown in Supplementary Materials.

Next, we aimed to elucidate the mechanisms associated with Nur77-medaited allergic reactions. miRNAs have been shown to play essential roles in allergic inflammations [15,29,30,31]. The 3′ UTR of Nur77 contains binding sites for various miRNAs, including miR-21a and miR-124 (Figure 6A). Antigen stimulation decreased the expressions of both miR-21a and miR-124 in RBL2H3 cells (Figure 6B). Nur77 negatively regulated the expressions of miR-21a and miR-124 in antigen-stimulated RBL2H3 cells (Figure 6B). miR-21a mimic exerted a negative effect on the increase in β-hexosaminidase activity induced by antigen stimulation in a dose-dependent manner in RBL2H3 cells (Figure 7A). Further, miR-21a mimic decreased the expression of Nur77 at the transcriptional level in RBL2H3 cells (Figure 7B). Moreover, miR-21a mimic exerted a negative effect on the increased expressions of NUR77 and MCP1 induced by antigen stimulation in RBL2H3 cells (Figure 7C). miR-124 mimic was found to prevent antigen from increasing β-hexosaminidase activity in RBL2H3 cells (Figure 7D). miR-124 mimic also prevented antigen from increasing the expression of NUR77 and MCP1 in RBL2H3 cells (Figure 7E). Thus, Nur77 is shown to regulate allergic reactions by forming a negative feedback loop with miR-21a and miR-124. 

### 3.4. miR-21a Negatively Regulates Anaphylaxis

**Figure 8:**
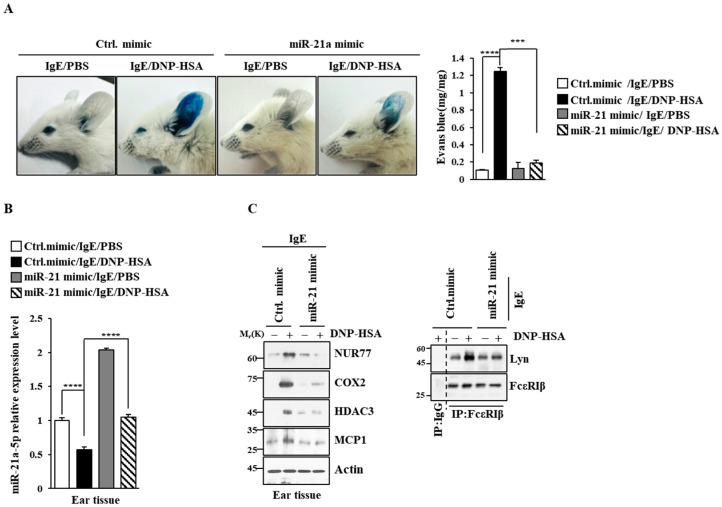
miR-21a mimic negatively regulates PCA. (**A**) BALB/C mice were given an intradermal injection of IgE (0.5 mg/kg) and an intravenous injection of the indicated mimic (each at 3 μg/kg). The next day, mice were intravenously injected with PBS or DNP-HSA (250 μg/kg) along with 2% (*v*/*v*) Evans blue solution. Each experimental group comprised four BALB/C mice. β-hexosaminidase activity assays employing tissue lysates were performed. The means ± S.E. of three independent experiments are shown. One-way ANOVA was carried out. ***, *p* < 0.001; ****, *p* < 0.0001. (**B**) QRT-PCR employing ear tissue lysates was performed. The means ± S.E. of three independent experiments are shown. One-way ANOVA was carried out. ****, *p* < 0.0001. (**C**) Immunoblot and immunoprecipitation employing ear tissue lysates were performed. Representative blots of three independent experiments are shown. Immunoprecipitation using isotype-matched IgG is shown. The uncropped blots are shown in Supplementary Materials.

**Figure 9:**
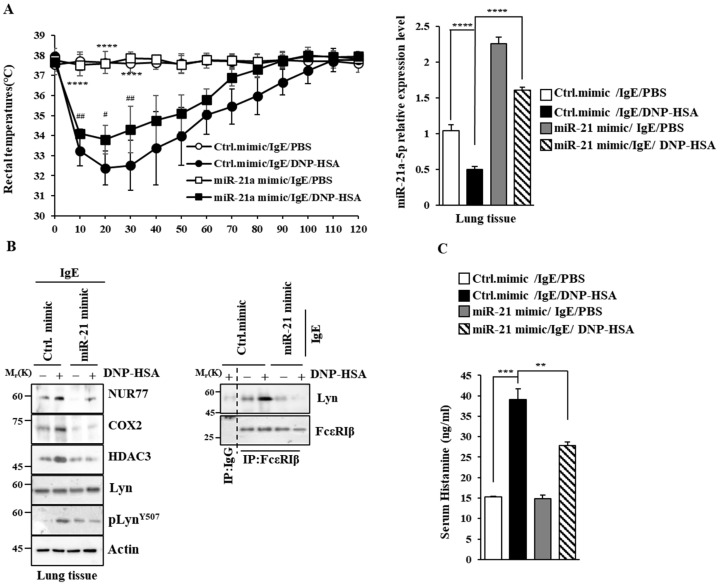
miR-21a mimic negatively regulates PSA. (**A**) BALB/C mice were given an intravenous injection of DNP-specific IgE (0.5 mg/kg) and the indicated mimic (each at 3 μg/kg). The next day, mice were intravenously injected with PBS or DNP-HSA (250 μg/kg), and their rectal temperatures were measured. Each experimental group comprised four BALB/C mice. QRT-PCR employing tissue lysates was performed. The means ± S.E. of three independent experiments are shown. One-way ANOVA was carried out. ****, *p* < 0.0001, comparison was made between Ctrl. mimic/IgE/PBS and Ctrl. mimic/IgE/DNP-HSA. #, *p* < 0.05; ##, *p* < 0.01, comparison was made between miR-21a mimic/IgE/DNP-HSA and Ctrl. mimic/IgE/DNP-HSA. (**B**) Immunoblot and immunoprecipitation employing lung tissue lysates were performed. Representative blots of three independent experiments are shown. Immunoprecipitation using isotype-matched IgG is shown. The uncropped blots are shown in Supplementary Materials. (**C**) Serum histamine level was determined as described. The means ± S.E. of three independent experiments are shown. One-way ANOVA was carried out. **, *p* < 0.01; ***, *p* < 0.001.

Next, we examined the effect of miR-21a on anaphylaxis. miR-21a mimic exerted a negative effect on the increase in vascular permeability seen in PCA (Figure 8A). miR-21a was shown to be decreased by antigen stimulation in PCA (Figure 8B). miR-21a mimic exerted a negative effect on the increased expression of NUR77 and the hallmarks of allergic reactions, such as COX2, HDAC3, and MCP1, in PCA (Figure 8C). Further, miR-21a mimic decreased the binding of FcεRI to LYN (Figure 8C). Moreover, miR-21a mimic suppressed the decrease in rectal temperatures in PSA (Figure 9A). The expression of miR-21a was shown to be decreased as a result of antigen stimulation in PSA (Figure 9A). miR-21a mimic was found to decrease the expressions of NUR77, COX2, HDAC3, and pLYN^Y507^ in PSA (Figure 9B). miR-21a mimic also decreased the binding of FcεRI to LYN (Figure 9B). miR-21a mimic additionally decreased the serum histamine level in PSA (Figure 9C). It is probable that the Nur77-miR-21a negative feedback loop may regulate anaphylaxis. 

### 3.5. C-Jun Regulates the Expression of Nur77

**Figure 10:**
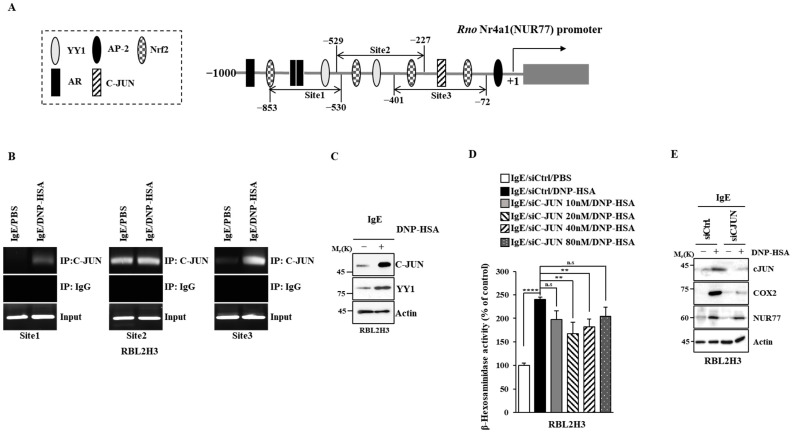
C-JUN increases the expression of Nur77. (**A**) Potential binding sites for transcription factors in the promoter sequences of Nur77. (**B**) The IgE-sensitized RBL2H3 cells were treated with DNP-HSA (100 ng/mL) for 1 h, followed by ChIP assays. Representative results of three independent experiments are shown. (**C**) Same as (**B**) except that immunoblot was performed. Representative blots of three independent experiments are shown. The uncropped blots are shown in Supplementary Materials. (**D**) The indicated siRNA was transfected into RBL2H3 cells. At 24 h after transfection, cells were sensitized with DNP-specific IgE (100 ng/mL) for another 24 h, followed by stimulation with DNP-HSA (100 ng/mL) for 1 h. β-hexosaminidase activity assays were performed. The means ± S.E. of three independent experiments are shown. One-way ANOVA was carried out. **, *p* < 0.01; ****, *p* < 0.0001. n.s. denotes not significant. (**E**) The indicated siRNA (each at 20 nM) was transfected into RBL2H3 cells. At 24 h after transfection, cells were sensitized with DNP-specific IgE (100 ng/mL) for another 24 h, followed by stimulation with DNP-HSA (100 ng/mL) for 1 h. Immunoblot was performed. Representative blots of three independent experiments are shown. The uncropped blots are shown in Supplementary Materials.

Next, the mechanism by which the expression of Nur77 is regulated was examined. Promoter sequences of Nur77 showed binding sites for transcription factors such as yin yang 1 (YY1), C-JUN, adaptor protein compkex-2 (AP-2), androgen receptor (AR), and neurofibromatosis type 2 (NF-2) (Figure 10A). Antigen increased the binding of C-JUN to the promoter sequences of Nur77 in RBL2H3 cells (Figure 10B). The expressions of c-JUN and YY1 were found to be increased by antigen stimulation in RBL2H3 cells (Figure 10C). The downregulation of c-Jun exerted a negative effect on the increase in β-hexosaminidase activity induced by antigen in RBL2H3 cells (Figure 10D). The downregulation of c-Jun also exerted a negative effect on the increased expression of COX2 and NUR77 by antigen in RBL2H3 cells (Figure 10E). Thus, c-Jun increases the expression of Nur77 to mediate allergic reactions. 

## 4. Discussion

RNA sequencing analysis revealed that Egr2 and Egr3 were increased by antigen stimulation in BMMCs (Figure 1A). Egr2 contributes to the pro-allergic (pro-asthmatic) properties of bone marrow-derived dendritic cells by serving as a target of miR-106b [32]. Ovalbumin-induced asthma has been shown to be associated with an increased expression of early growth response 1 (Egr-1) [33]. This implies that Egr1 plays a role in allergic inflammations such as anaphylaxis. Antigen stimulation increased the expression of Egr1 in RBL2H3 cells (Figure 2). 

The expression of Nor1 was found to be increased upon antigen stimulation in BMMCs (Figure 1). Prior studies have shown that the polarization of M2 macrophages occurs during allergic inflammation [7,8,34]. Allergic inflammation is accompanied by cellular interactions, and it involves increased autophagic flux [7,35]. The polarization of M2 macrophages can increase the expression of NOR1 in humans [36]. This implies that Nor1 plays a role in anaphylaxis.

Nur77 and Nurr1 were found to be upregulated in antigen-stimulated BMMCs (Figure 1A). Nur77 and Nurr1 increase the expression of TNF in antigen-stimulated BMMCs [37]. Antigen increases the expressions of both TH2 cytokine and TNF in BMMCs [38]. Histamine-induced angiogenesis is known to be mediated by Nur77 [39]. Histamine plays an essential role in anaphylaxis [28]. Nur77 can regulate inflammatory responses mainly by suppressing NF-κB activation [40]. Cigarette smoke-induced airway inflammation involves a decreased expression of Nur77 [40]. The downregulation of Nur77 is known to exacerbate airway inflammation [40]. In the current study, we found that Nur77 was necessary for anaphylaxis (Figure 4 and Figure 5).

Genes that are regulated by Nur77 may play essential roles in allergic inflammations. Promoter analysis of Nur77 showed potential binding sites for transcription factors such as C-JUN, YYI, FosB, and nuclear factor erythroid 2-related factor 2 (NRF2) (personal observations). Exogenous cholesterol incorporated into lipid rafts can induce mast cell activation by increasing the expression of FosB [41]. In this study, we showed the binding of c-JUN to the promoter sequences of Nur77.

Nur77 can promote the polarization of M2 macrophages and inhibit autophagic cell death during breast cancer progression [42]. Autophagy can mediate atopic dermatitis by promoting the polarization of M2 macrophages [43]. Previous research has shown that the polarization of M2 macrophages occurs during allergic inflammations [8]. Thus, Nur77 might promote autophagic flux during allergic inflammations. Since allergic inflammations involve cellular interactions [7,8,44], Nur77 may mediate anaphylaxis by promoting cellular interactions. Nur77 is known to mediate the angiogenic effects of vascular endothelial growth factor-A (VEGF-A) and prostaglandin E (PGE) [45]. Culture medium of antigen-stimulated mast cells might display an angiogenic effect and induce the polarization of M2 macrophages in a Nur77-dependent manner. The targets of Nur77 may mediate allergic reactions. These genes could thus serve as targets for developing anti-allergy drugs.

Resveratrol can bind to NUR77 and act as an anticancer agent [46]. Resveratrol can decrease the expression of TSLP, which is a marker of atopic dermatitis [47]. Resveratrol can suppress human mast cell degranulation by inhibiting the phosphorylation of extracellular-regulated kinase (ERK) [48]. Resveratrol has previously been shown to protect against mast cell-driven skin inflammation by inhibiting sphingosine signaling in association with signal transducer and activator of transcription 3 (STAT3) and NF-kB [49]. Resveratrol also inhibits mast cell activation by regulating NRF2 [50]. Resveratrol also decreases the expression of MCP1, which is a hallmark of allergic reactions [51]. Altogether, these reports suggest that resveratrol may suppress atopic dermatitis, asthma, and anaphylaxis. Identifying targets of resveratrol may therefore be helpful for achieving a better understanding of the mechanism associated with anaphylaxis.

## 5. Conclusions

Nur77 plays essential roles in lung diseases such as asthma, acute lung injury, and pulmonary fibrosis [52]. In this study, we show the roles of Nur77 and miR-21a in anaphylaxis. To elucidate the mechanisms of Nur77-promoted anaphylaxis, it is necessary to identify that Nur77 molecular networks comprise mRNAs, miRNAs, and cytokines. Nur77 molecular networks can provide clues that can be used in the development of anti-allergy drugs.

## Data Availability

All data are available upon request to the corresponding author.

## Supplementary Materials

The following supporting information can be downloaded at: https://www.mdpi.com/article/10.3390/cimb46040199/s1, Table S1: The sequences of microRNA mimics. Table S2: The sequences of SiRNAs. Table S3: Primer sequences for qRT-PCR.
