# Supplementary material for: Nur77 Mediates Anaphylaxis by Regulating miR-21a
Source: Curr Issues Mol Biol. 2024 Apr 6;46(4):3175–92. doi: 10.3390/cimb46040199 (PMC11048962; doi:10.3390/cimb46040199)
Supplement: Supplementary file 1 [file cimb-46-00199-s001.zip › cimb-2904401-supplementary.pdf]

Supplementary Table S1. The sequences of microRNA mimics

Name	Company	Sequence (5'-3')
Negative control	Bioneer	AccuTarget™ miRNA Negative Control, mimic #1 (Cat. SMC-2002)
miR-21	Bioneer	UAGCUUAUCAGACUGAUGUUGA
miR-124	Bioneer	CGUGUUCACAGCGGACCUUGAU

Supplementary Table S2. The sequences of SiRNAs

Name	Company	Sequence (5'-3')
Negative control	Bioneer	AccuTarget™ Negative Control siRNA (Cat. SN-1002)
SiNR4A1(Rat)	Bioneer	Sense: GAACCGCAUUGCUAGCUGUtt<br>Antisense: ACAGCUAGCAAUGCGGUUCtg

Supplementary Table S3. Primer sequences for qRT-PCR

Name	Sequence (5'-3')
Fosb (Rat)	F: GATCCCTTACGAAGAGGGGC<br>R: CGTCTTCCTTAGCGGATGTTG
Nab2 (Rat)	F: CAGCGCATGGGCTATGGA<br>R: CCCACACGATCATGGGAGAC
EGR1 (Rat)	F: AACACTTTGTGGCCTGAACC<br>R: GAGGCAGAGGAAGACGATGA
NR4A1 (Mouse)	F: GCGTCGGCTTCCTTTAAGTT<br>R: AATCAGAGCCACTGGAGGAC
NR4A1 (Rat)	F: GCCCATTAGACGAGACCCTG<br>R: TGAACCATCCCAAGGGGAGA
NR4A2 (Rat)	F: GTCTGTGGAGACAACGCTTC<br>R: AGGCAGATGTACTTGGCACT

NR4A3<br>(Rat)	F: TCCGCCTTCTACACCGA<br>R: GCCTTCATCTGCGGGTC

Raw Data

Immunoblot

Figure 3A

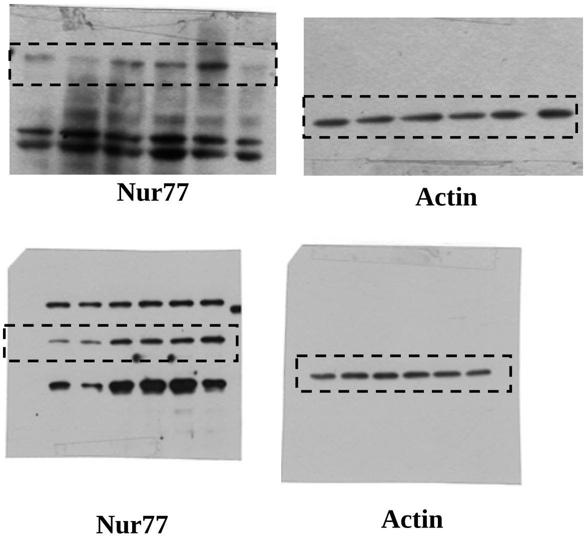

Figure 3C

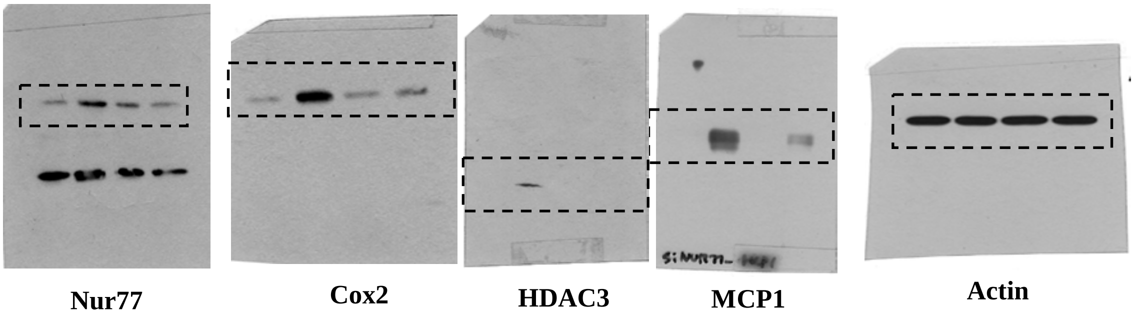

Figure 4C

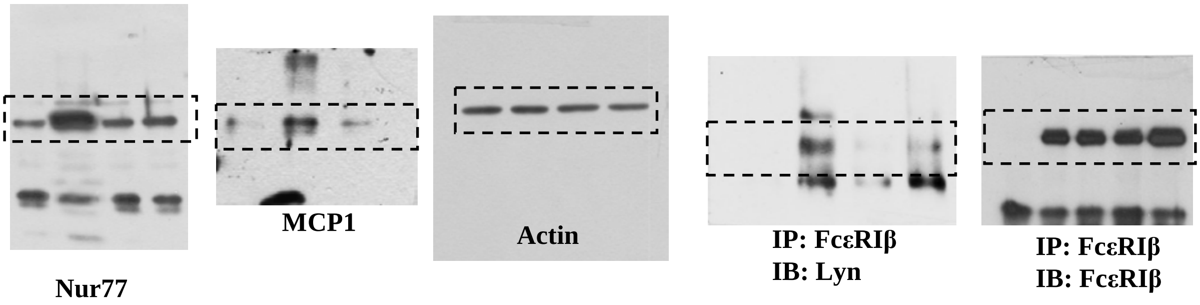

Figure 5C

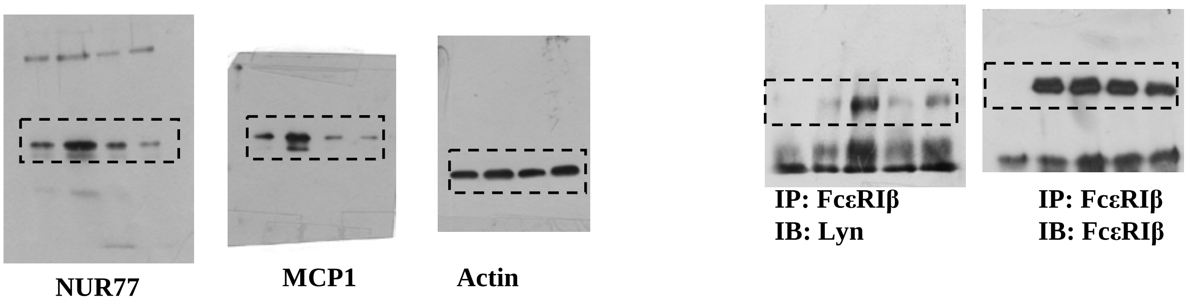

Figure 7C

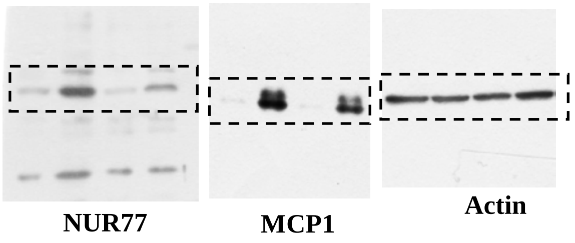

Figure 7E

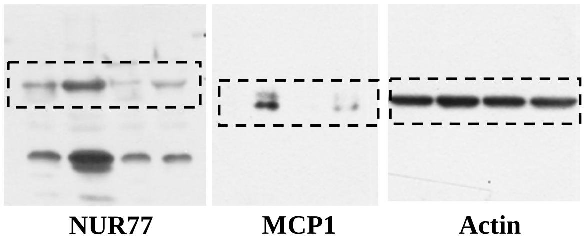

Figure 8C

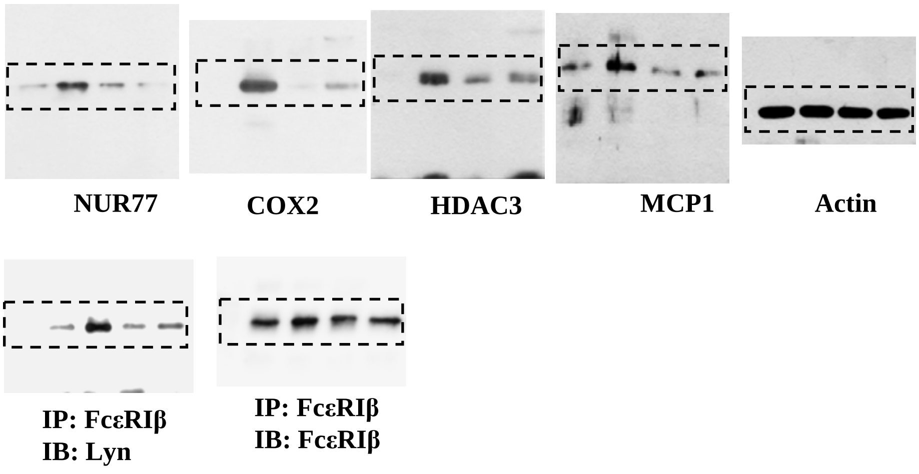

Figure 9B

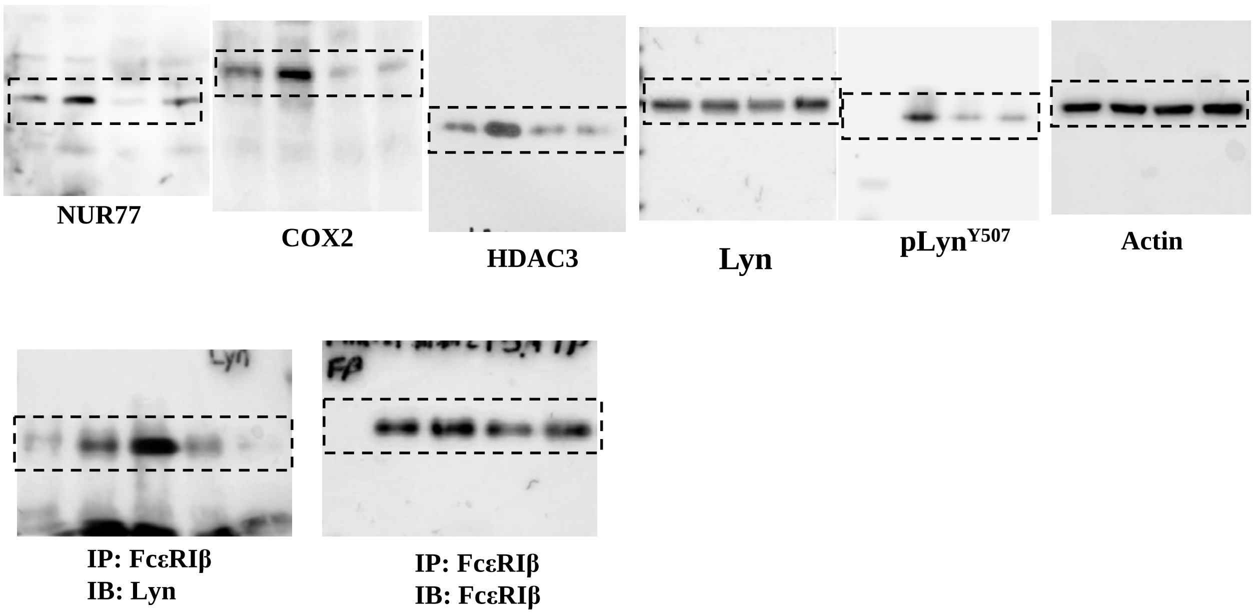

Figure 10C

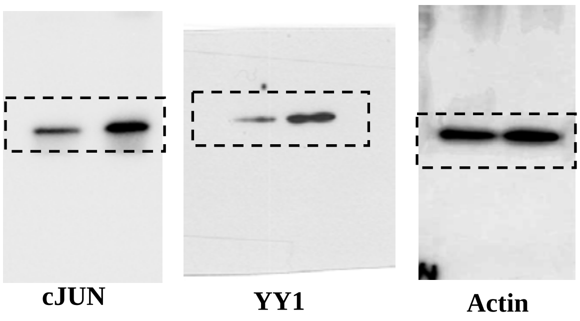

Figure 10E

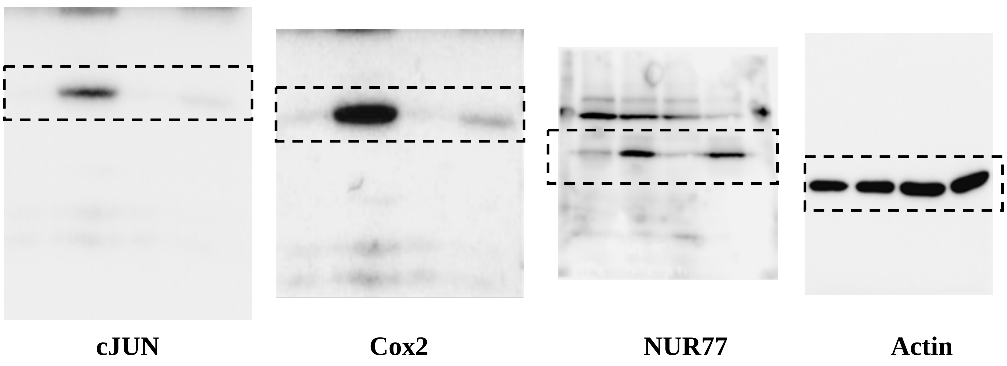

PCA

Figure 4A

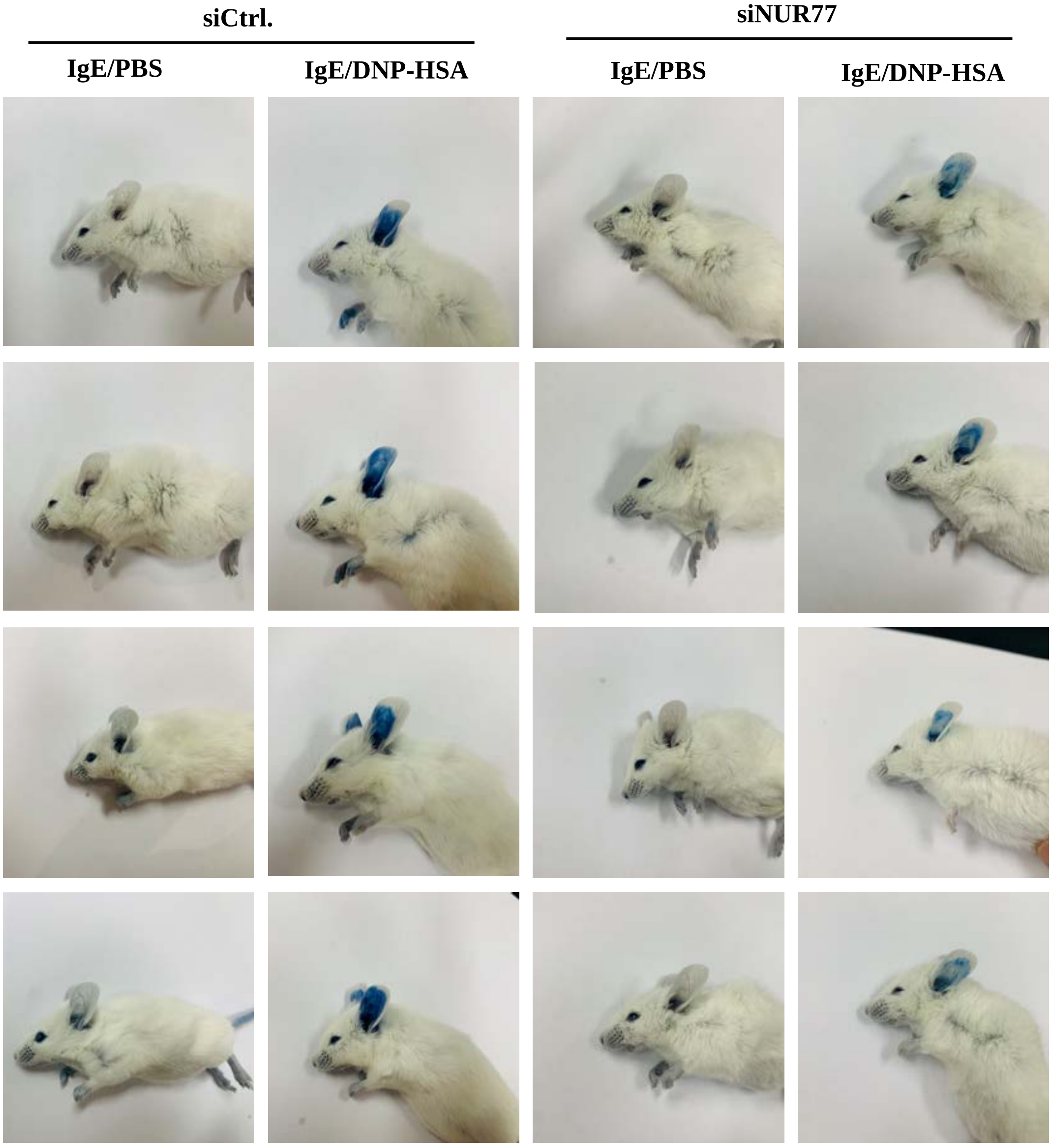

Figure 8A

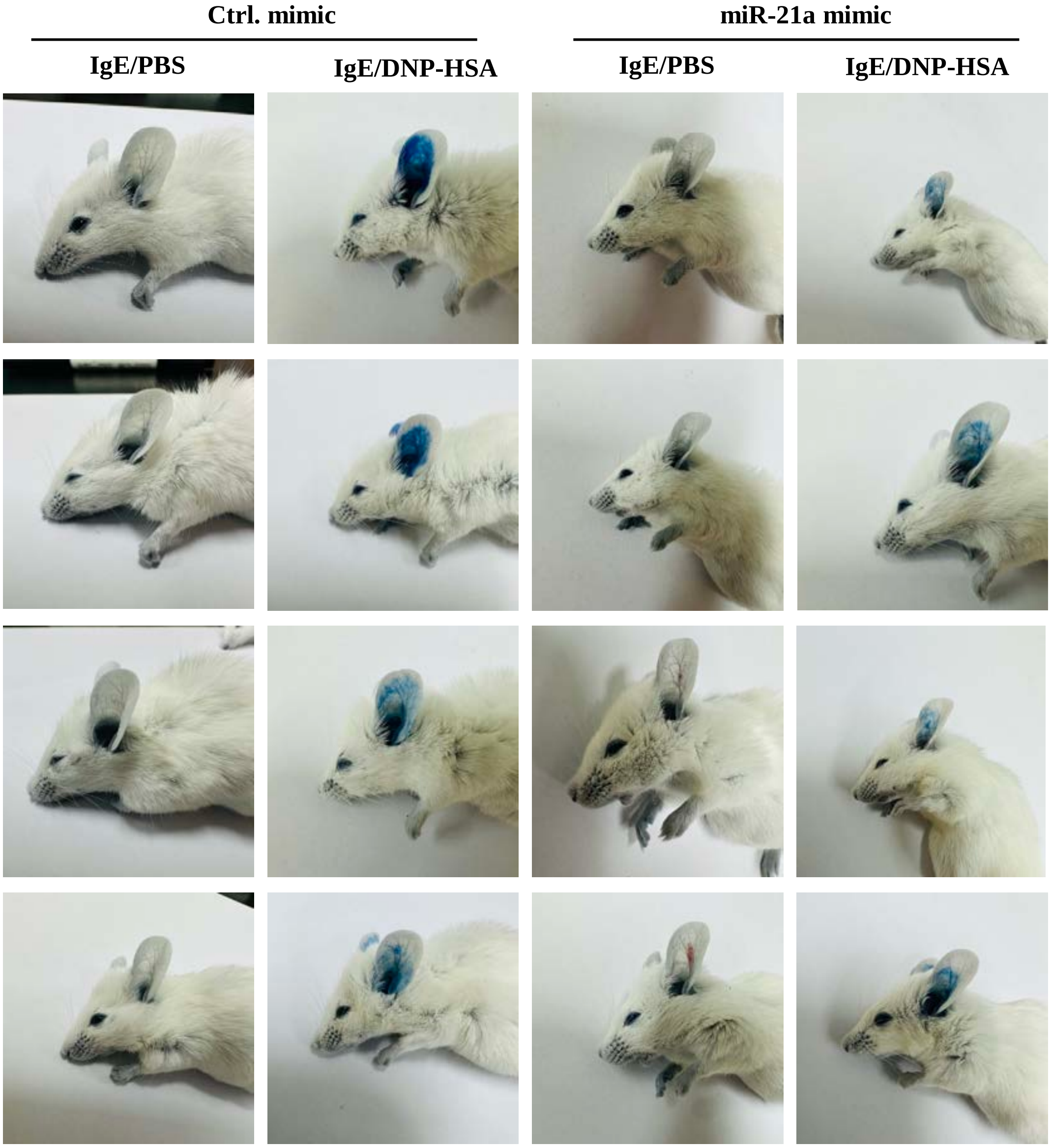
Ctrl. mimic
miR-21a mimic
IgE/PBS
IgE/DNP-HSA
IgE/PBS
IgE/DNP-HSA

ChIP assay

Figure 10B

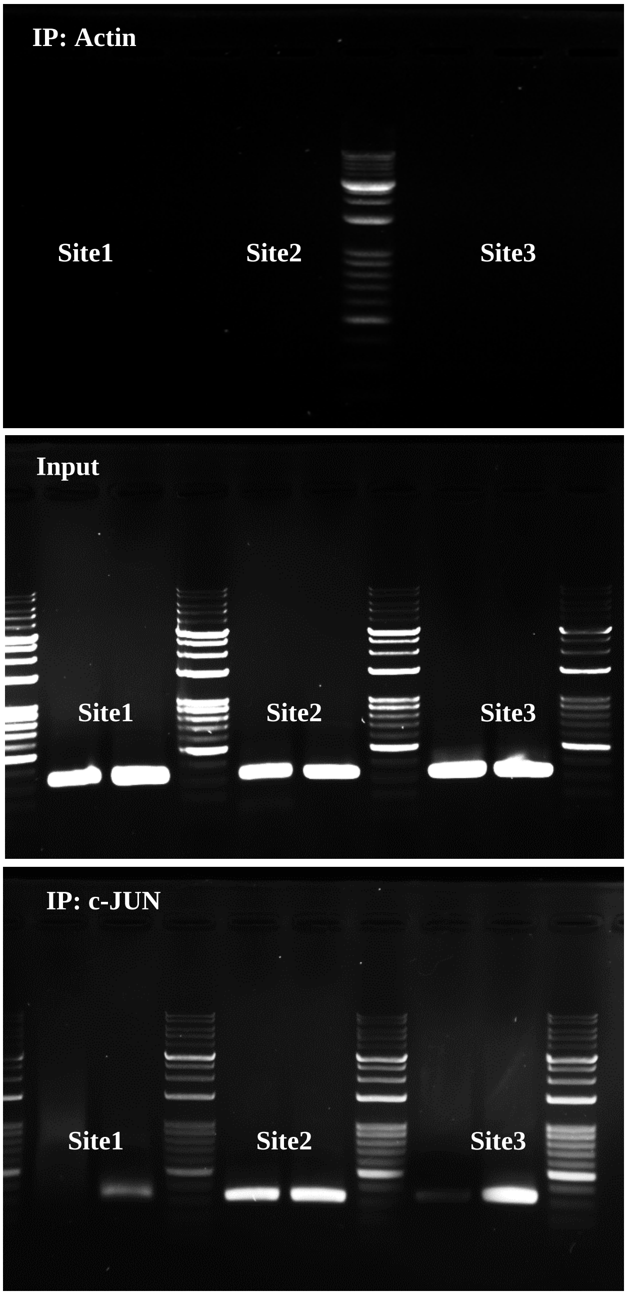

qRT-PCR

Fig.2 RBL2H3_DNP-HSA

Target gene \ Sample	IgE/Mock	IgE/DNP-HSA
**NR4A1**	1.075129448	3.20630861
	0.92670716	3.292076143
	0.998163392	2.63893442
**NR4A2**	1.117192824	2.277940325
	0.941038296	2.172283304
	0.941768879	1.945778237
**NR4A3**	0.790171233	2.598021806
	0.813715078	2.367708286
	1.396113689	2.235275169
**EGR1**	1.030257432	1.680124448
	0.99988887	2.015121298
	0.969853698	1.792234289
**FosB**	0.908850175	5.360858928
	1.161762165	5.328440134
	0.92938766	5.702809235
**Nab2**	0.957300878	1.70298678
	1.083863082	1.741497172
	0.958836041	1.676464633

Fig.3B RBL2H3_siNUR77

Target gene \ Sample	siCtrl./IgE/PBS	siCtrl./IgE/DNP-HSA	siNUR77/IgE/PBS	siNUR77/IgE/DNP-HSA
**NR4A1**	1.119009475	2.710196934	0.74844424	0.771132782
	1.075008131	2.695499725	0.595833659	0.749304278
	0.805982393	2.466342589	0.636121523	0.780528123

Fig.4B PCA_siNUR77

Target gene \ Sample	siCtrl./IgE/PBS	siCtrl./IgE/DNP-HSA	siNUR77/IgE/PBS	siNUR77/IgE/DNP-HSA
**NR4A1**	0.99772554	2.756831528	1.232222832	1.138212401
	1.035938907	2.776924921	1.060647337	1.327270884
	0.96750845	2.840505512	1.125232089	1.568419391

Fig.5B PSA_siNUR77

Target gene \ Sample	siCtrl./IgE/PBS	siCtrl./IgE/DNP-HSA	siNUR77/IgE/PBS	siNUR77/IgE/DNP-HSA
**NR4A1**	0.974211408	1.520139774	0.703308823	0.820576669
	1.025788592	1.538370013	0.789353623	0.819788353

Fig.6B RBL2H3_siNUR77

Target gene \ Sample	siCtrl./IgE/PBS	siCtrl./IgE/DNP-HSA	siNUR77/IgE/PBS	siNUR77/IgE/DNP-HSA
**miR-21a-5p**	2.619293919	1.044515647	3.966458517	4.227775689
	2.521668933	1.010504011	3.836346071	4.112022982
	2.497217912	0.94742973	4.016404339	3.993294782
**miR-124-3p**	5.437273849	0.899251566	10.35437936	9.68824869
	4.99742568	1.013925395	9.51198213	9.781803038
	5.597372628	1.096763015	10.64192719	10.40757966

Fig.7B RBL2H3_miR-21 mimic

Sample / Target gene	Ctrl.mimic/IgE/PBS	Ctrl.mimic/IgE/DNP-HSA	miR-21 mimic/IgE/PBS	miR-21 mimic/IgE/DNP-HSA
**miR-21a-5p**	1.002687855	1.581434608	28.39263248	18.86530588
	0.978405615	1.669026656	31.86302401	17.45443773
	1.01933118	1.745763106	28.00029007	15.3816203
**NR4A1**	1.061244726	1.433928595	0.368369502	0.357017302
	0.969377637	1.337902687	0.212084209	0.361013446
	0.969377637	1.418338316	0.426181902	0.303129742

Fig.8B PCA_miR-21 mimic

Sample / Target gene	Ctrl.mimic/IgE/PBS	Ctrl.mimic/IgE/DNP-HSA	miR-21 mimic/IgE/PBS	miR-21 mimic/IgE/DNP-HSA
**miR-21a-5p**	0.974461439	0.598899961	2.025432437	1.019030927
	0.984100262	0.517539638	2.067901841	1.041266328
	1.042787927	0.587248015	2.020457903	1.09279455

Fig.9A PSA_miR-21 mimic

Sample / Target gene	Ctrl.mimic/IgE/PBS	Ctrl.mimic/IgE/DNP-HSA	miR-21 mimic/IgE/PBS	miR-21 mimic/IgE/DNP-HSA
**miR-21a-5p**	0.961436004	0.508301259	0.7294247	0.747038153
	0.955216433	0.438459666	0.791825492	0.781958621
	1.088874514	0.497623181	0.776844121	0.785775416

beta-hexosaminidase activity

Fig.3B RBL2H3_siNR4A1

siCtrl./IgE/PBS	siCtrl./IgE/DNP-HAS	10nM siNR4A1/IgE/DNP-HSA	20nM siNR4A1/IgE/DNP-HSA	40nM siNR4A1/IgE/DNP-HSA	80nM siNR4A1/IgE/DNP-HSA
100	360	222.8571429	255.7142857	259.2857143	277.8571429
107.1428571	330.7142857	219.2857143	235.7142857	260	242.1428571
100	355.7142857	254.2857143	250	274.2857143	280.7142857

Fig.7A RBL2H3_miR-21 mimic

Ctrl.mimic/IgE/PBS	Ctrl.mimic/IgE/DNP-HSA	0nM miR-21 mimic/IgE/DNP-HS	20nM miR-21 mimic/IgE/DNP-HS	40nM miR-21 mimic/IgE/DNP-HSA
82.94930876	268.8940092	136.8663594	141.7050691	143.7788018
117.5115207	281.3364055	133.4101382	127.1889401	183.1797235
99.53917051	243.3179724	125.8064516	180.4147465	167.9723502

Fig.7D RBL2H3_miR-124-1 mimic

Ctrl.mimic/IgE/PBS	Ctrl.mimic/IgE/DNP-HSA	nM miR-124-1mimic/IgE/DNP-H	0nM miR-124-1mimic/IgE/DNP-HS	40nM miR-124-1mimic/IgE/DNP-HSA	80nM miR-124-1mimic/IgE/DNP-HSA
97.6744186	205.1162791	106.0465116	126.9767442	113.7209302	131.8604651
104.6511628	246.2790698	117.2093023	132.5581395	114.4186047	96.97674419
97.6744186	207.9069767	98.37209302	106.744186	121.3953488	134.6511628

Fig.10D RBL2H3_siCJUN

siCtrl./IgE/PBS	siCtrl./IgE/DNP-HAS	10nM siCJUN/IgE/DNP-HAS	20nM siCJUN/IgE/DNP-HSA	40nM siCJUN/IgE/DNP-HSA	80nM siCJUN/IgE/DNP-HSA
101.1363636	251.1363636	184.0909091	153.4090909	186.3636364	196.5909091
100	242.0454545	209.0909091	167.0454545	172.7272727	203.4090909
109.0909091	251.1363636	220.4545455	200	205.6818182	232.9545455

Evansblue absorbance

Fig.4A PCA_siNUR77

siCtrl./IgE/PBS	siCtrl./IgE/DNP-HSA	siNUR77/IgE/PBS	siNUR77/IgE/DNP-HSA
1.14614	31.14798	2.821	27.98031
1.51024	46.00326	2.0928	8.53737
4.13176	53.57654	3.14869	12.54247
8.53737	45.23865	4.05894	7.37225

Fig.8 PCA_miR-21 mimic

Ctrl.mimic/IgE/PBS	Ctrl.mimic/IgE/DNP-HSA	miR-21 mimic/IgE/PBS	miR-21 mimic/IgE/DNP-HSA
0.097612	1.15600625	0.1437495	0.175123
0.11883525	1.241822	0.086539	0.234179
0.10960775	1.12094175	0.0662385	0.2064965
0.10591675	1.11725075	0.252634	0.1400585

Serum Histamine ELISA

Fig.5 PSA_siNUR77

siCtrl./IgE/PBS	siCtrl./IgE/DNP-HSA	siNUR77/IgE/PBS	siNUR77/IgE/DNP-HSA
8.900713799	19.39855292	7.648027457	9.64639862
9.047765805	15.73530441	7.221996246	6.722037437

Fig.9 PSA_miR-21 mimic

Ctrl.mimic/IgE/PBS	Ctrl.mimic/IgE/DNP-HSA	miR-21 mimic/IgE/PBS	miR-21 mimic/IgE/DNP-HSA
15.25695213	37.11002668	14.35351073	27.13684996
15.45111559	40.90434139	15.45111559	28.40333769
